# The Phenotypic Continuum of *ATP1A3*-Related Disorders

**DOI:** 10.1212/WNL.0000000000200927

**Published:** 2022-10-04

**Authors:** Aikaterini Vezyroglou, Rhoda Akilapa, Katy Barwick, Saskia Koene, Catherine A. Brownstein, Muriel Holder-Espinasse, Andrew E. Fry, Andrea H. Németh, George K. Tofaris, Eleanor Hay, Imelda Hughes, Sahar Mansour, Santosh R. Mordekar, Miranda Splitt, Peter D. Turnpenny, Demetria Demetriou, Tamara T. Koopmann, Claudia A.L. Ruivenkamp, Pankaj B. Agrawal, Lucinda Carr, Virginia Clowes, Neeti Ghali, Susan Elizabeth Holder, Jessica Radley, Alison Male, Sanjay M. Sisodiya, Manju A. Kurian, J. Helen Cross, Meena Balasubramanian

**Affiliations:** From the Developmental Neurosciences (A.V., K.B., M.A.K., J.H.C.), UCL Great Ormond Street Institute of Child Health, London, UK; Department of Neurology (A.V., L.C., M.A.K., J.H.C.), Great Ormond Street Hospital, London, UK; Department of Clinical Genetics (R.A., M.H.-E.), Guy's and St Thomas' NHS Foundation Trust, Guy's Hospital, London, United Kingdom; Department of Clinical Genetics (S.K., T.T.K., C.A.L.R.), Leiden University Medical Center, The Netherlands; Division of Genetics and Genomics (C.A.B., P.B.A.), the Manton Center for Orphan Disease Research, Boston Children's Hospital, MA; Department of Pediatrics (C.A.B., P.B.A.), Harvard Medical School, Boston, MA; All Wales Medical Genomics Service (A.E.F.), NHS Wales Cardiff and Vale University Health Board, Institute of Medical Genetics, University Hospital of Wales, UK; Division of Cancer and Genetics (A.E.F.), School of Medicine, Cardiff University, UK; Nuffield Department of Clinical Neurosciences (A.H.N., G.K.T.), University of Oxford, UK; Department of Clinical Genetics (E.H., A.M.), Great Ormond Street Hospital, London, UK; Department of Paediatric Neurology (I.H.), Central Manchester University Hospitals NHS Foundation Trust, UK; SW Thames Regional Genetics Service (S.M.), St George's University Hospitals NHS Foundation Trust, UK; Department of Paediatric Neurology (S.R.M.), Ryegate Children's Centre, Sheffield Children's Hospital, United Kingdom; Institute of Genetic Medicine (M.S.), Newcastle Upon Tyne, UK; Clinical Genetics (P.D.T.), Royal Devon & Exeter NHS Foundation Trust, UK; Aneurin Bevan University Health Board (D.D.), Royal Gwent Hospital, Newport, UK; Division of Newborn Medicine (P.B.A.), Boston Children's Hospital, MA; North West Thames Regional Genetics Service (V.C., N.G., S.E.H., J.R.), Northwick Park Hospital, Middlesex, UK; Department of Clinical and Experimental Epilepsy (S.M.S.), UCL Queen Square Institute of Neurology, London, UK; Department of Oncology & Metabolism (M.B.), University of Sheffield, UK; and Sheffield Clinical Genetics Service (M.B.), Sheffield Childrens NHS Foundation Trust, UK.

## Abstract

**Background and Objectives:**

*ATP1A3* is associated with a broad spectrum of predominantly neurologic disorders, which continues to expand beyond the initially defined phenotypes of alternating hemiplegia of childhood, rapid-onset dystonia parkinsonism, and cerebellar ataxia, areflexia, pes cavus, optic atrophy, sensorineural hearing loss syndrome. This phenotypic variability makes it challenging to assess the pathogenicity of an *ATP1A3* variant found in an undiagnosed patient. We describe the phenotypic features of individuals carrying a pathogenic/likely pathogenic *ATP1A3* variant and perform a literature review of all *ATP1A3* variants published thus far in association with human neurologic disease. Our aim is to demonstrate the heterogeneous clinical spectrum of the gene and look for phenotypic overlap between patients that will streamline the diagnostic process.

**Methods:**

Undiagnosed individuals with *ATP1A3* variants were identified within the cohort of the Deciphering Developmental Disorders study with additional cases contributed by collaborators internationally. Detailed clinical data were collected with consent through a questionnaire completed by the referring clinicians. PubMed was searched for publications containing the term “ATP1A3” from 2004 to 2021.

**Results:**

Twenty-four individuals with a previously undiagnosed neurologic phenotype were found to carry 21 *ATP1A3* variants. Eight variants have been previously published. Patients experienced on average 2–3 different types of paroxysmal events. Permanent neurologic features were common including microcephaly (7; 29%), ataxia (13; 54%), dystonia (10; 42%), and hypotonia (7; 29%). All patients had cognitive impairment. Neuropsychiatric diagnoses were reported in 16 (66.6%) individuals. Phenotypes were extremely varied, and most individuals did not fit clinical criteria for previously published phenotypes. On review of the literature, 1,108 individuals have been reported carrying 168 different *ATP1A3* variants. The most common variants are associated with well-defined phenotypes, while more rare variants often result in very rare symptom correlations, such as are seen in our study. Combined Annotation-Dependent Depletion (CADD) scores of pathogenic and likely pathogenic variants were significantly higher and variants clustered within 6 regions of constraint.

**Discussion:**

Our study shows that looking for a combination of paroxysmal events, hyperkinesia, neuropsychiatric symptoms, and cognitive impairment and evaluating the CADD score and variant location can help identify an *ATP1A3*-related condition, rather than applying diagnostic criteria alone.

Throughout the past 20 years, pathogenic variants in *ATP1A3* have been discovered to cause an ever-expanding range of rare neurologic phenotypes, affecting both children and adults. *ATP1A3* encodes the α3 subunit of a sodium-potassium-ATPase (NKA) present in excitable (neuronal and cardiac) cells. The α3-subunit has a relatively low Na+ affinity coupled with a high affinity to adenosine triphosphate (ATP).^1,2^ Consequently, the NKA carrying the α3-subunit is ideally configured to clear high intraneuronal sodium concentrations occurring after intense neuronal firing, by being able to use the low concentration of ATP that will occur near the neuronal membrane shortly after an energy-demanding task.

In 2004, variants in *ATP1A3* were linked to rapid-onset dystonia parkinsonism (RDP).^3^ In 2012 such variants were discovered to also be a cause of alternating hemiplegia of childhood (AHC)^4^. In 2014, the gene was linked to cerebellar ataxia, areflexia, pes cavus, optic atrophy, sensorineural deafness (CAPOS) syndrome.^5,6^ Since then, many more phenotypes, including early infancy epileptic encephalopathy (EIEE)^7^ sometimes accompanied by polymicrogyria (PMG),^8–10^ relapsing encephalopathy with cerebellar ataxia (RECA)^11,12^/fever-induced paroxysmal weakness and encephalopathy (FIPWE),^13^ childhood onset schizophrenia (COS),^14^ and D-DEMØ, a phenotype characterized by dystonia, facial dysmorphism encephalopathy, severe developmental delay, MRI abnormalities (including cerebellar hypoplasia), and lacking the AHC hallmark symptom of paroxysmal hemiplegia,^15^ were also attributed to *ATP1A3* variants. Table 1 summarizes phenotypes currently associated with *ATP1A3* variants. In a recent editorial, Salles et al.^16^ questioned the utility of describing evermore distinct phenotypes associated with *ATP1A3* mostly because some individuals do not fit any of the currently proposed phenotypes.

**Table 1:**
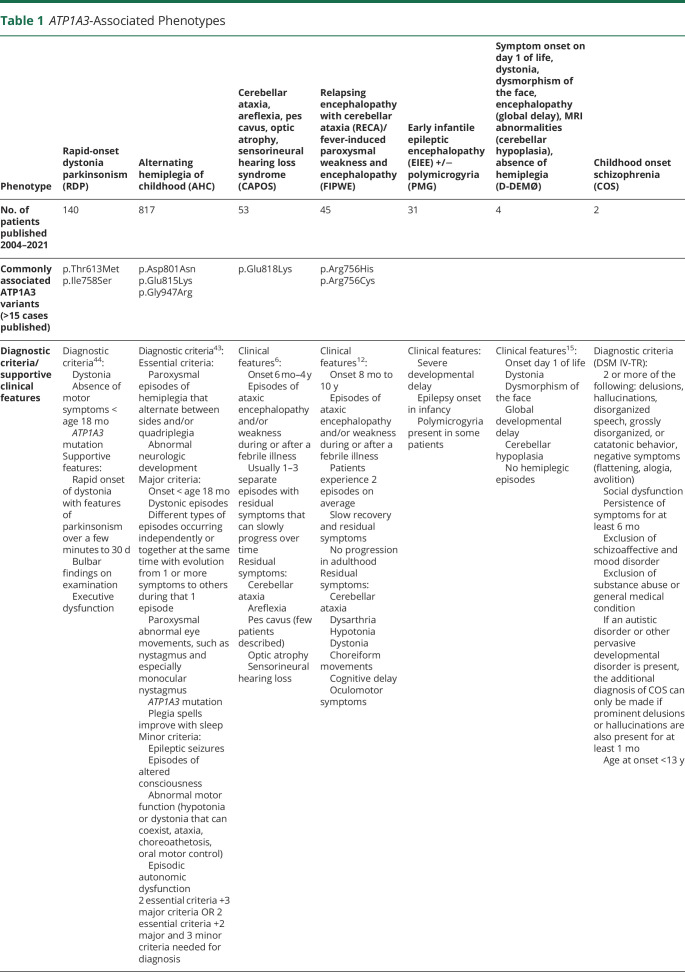
*ATP1A3*-Associated Phenotypes

In this study, we aim to describe the phenotypic features of a cohort of previously undiagnosed individuals with developmental delay and a neurologic presentation, carrying a pathogenic/likely pathogenic *ATP1A3* variant, and examine where they fit within the current spectrum of *ATP1A3*-related disorders. We also perform a literature review of all *ATP1A3* variants published thus far in association with human neurologic disease. Our work clearly demonstrates the heterogeneous clinical spectrum associated with *ATP1A3* variants, as well as phenotypic overlap between patients, that will streamline the diagnostic process.

## Methods

### Subject Cohort

An application was made to the Deciphering Developmental Disorders (DDD) study^17^ for a Complementary Analysis Project, allowing access to anonymized details of individuals with *ATP1A3* variants identified through this study.^18^ If variant analysis and phenotypic details made pathogenicity likely, responsible clinicians were contacted to invite patients and their families to study recruitment. Some of the contacted clinicians had further individuals with *ATP1A3* variants in their care, which they put forward as potential participants. Genomic diagnosis was reached through trio whole-exome sequencing (WES) for the DDD participants and through either WES or diagnostic gene panels for the other study participants. Phenotypic details were collected using a standardized clinical proforma covering all symptoms previously reported in *ATP1A3*-related disorders and MRI features.

We used the UpSetR package in R to visualize intersections of signs and symptoms, trying to identify common phenotypes among individuals. As the clinical proforma included a long list of symptoms and signs due to the phenotypic variability of *ATP1A3*-related conditions, we decided to also group symptoms into broader categories where possible. We formed 4 categories:

1. Paroxysmal events, including hemiplegic events, dystonic episodes, seizures, abnormal eye movements, apnoea, and autonomic episodes.
2. Persistent movement disorders, including hyperkinetic phenotypes (dystonia and chorea) and ataxia.
3. Cognitive impairment (mild, moderate, and severe).
4. Neuropsychiatric symptoms, including behavioral difficulties, attention-deficit and hyperactivity disorder (ADHD), autism spectrum disorder (ASD), and COS.

To evaluate the pathogenicity of missense variants, in silico prediction tools were used, including sorting tolerant from intolerant (SIFT),^19^ polymorphism phenotyping v2, PROVEAN,^20^ MutationTaster,^21^ and CADD scores.^22^ The presence of variants in the healthy population (141,456 individual exomes/genomes in the Genome Aggregation Database v2.1.1 [gnomAD^23^]) was determined and conservation across 14 species evaluated, as well as variant segregation within each family. A literature search was performed to determine whether variants were recurrent or previously reported. American College of Medical Genetics and Genomics (ACMG) classification criteria^24^ were used to assess pathogenicity. Individuals with class 4 or 5 variants (likely pathogenic or pathogenic variants based on ACMG variant classification, respectively) were included in this study. Informed consent was obtained from all patients and/or their guardians.

### Literature Review

A PubMed search was performed for all papers that included the term “*ATP1A3*” from January 2004, the year of the first association of an *ATP1A3* variant with human disease (RDP), to August 2021. Included in this study are all publications in English and 1 in Spanish language (where phenotypic and genomic information was included in the English abstract) reporting individuals carrying a heterozygous *ATP1A3* variant considered to be pathogenic. One publication in Japanese, 1 in Russian, and 3 in Chinese were not included. Only publications with sufficient details about the variant (nucleotide change and/or amino acid change and gene transcript) and patient phenotype were included. All individuals published were counted, unless clearly stated that they had already been published elsewhere, in which case they were only counted once. However, it is possible that cohorts overlap. Although *ATP1A3* variants have been reported in different gene transcripts, in this article, all variant nomenclatures adhere to transcript NM_152296 (isoform 1). Variants that were inconsistent with all available transcripts were presumed to have been reported incorrectly and excluded.

We calculated Combined Annotation-Dependent Depletion (CADD) scores^22^ for all missense *ATP1A3* variants collected from the literature and for all missense variants reported within ClinVar as likely benign and benign and compared them. Unlike other genomic annotations that tend to exploit a single information type (i.e., conservation), CADD is a framework that objectively integrates many diverse annotations into a single, quantitative score. The integrated annotations include conservation metrics, functional genomic data, transcript information, and protein-level scores (Grantham, SIFT, and PolyPhen). CADD calculates a raw score and a ‟PHRED-scaled” score. PHRED-scaled scores are normalized to all potential ∼9 billion SNVs, thus providing a comparable unit for analysis. Hence, a PHRED-scaled score of ≥10 indicates a raw score in the top 10% of all possible reference genome SNVs, a score of ≥20 or greater indicates a raw score in the top 1%, etc.^25^ The developers of CADD do not suggest a rigid cutoff to suggest pathogenicity; however, looking at various HGMD molecular categories of 174,183 disease-associated deleterious mutations, one study found mean CADD scores for pathogenic missense variants to be above 20.^26^ The tool is freely available on cadd.gs.washington.edu.

### Constraint Analysis

Constraint analysis^27^ was performed on all missense pathogenic variants in the study cohort and for published cases and compared with the reported benign missense variants in the population database GnomAD. The number of benign missense variants present within every 10 amino acid residues was plotted across the length of the gene, and from this, the missense constraint heat map included in Figure 4 was generated using the following parameters: dark green = >20 variants, light green = 11–20 variants, yellow = 8–10 variants, light red = 1–3 variants, and dark red = 0 variants. Locations of pathogenic missense variants identified in the study cohort were also plotted across the length of the gene, visually highlighting regions of the gene that are tolerant and intolerant to missense variation.

### Data Availability

All data relevant to our patient cohort are published within the text and tables of this article. The detailed data extracted from the literature to conduct the literature review may be shared at the request of any qualified investigator for purposes of replicating procedures and results.

### Standard Protocol Approvals, Registrations, and Patient Consents

The DDD study has UK Research Ethics Committee approval (10/H0305/83, granted by the Cambridge South REC, and GEN/284/12, granted by the Republic of Ireland REC). The “Natural history in *ATP1A3-*related disease: a deep phenotyping-genotyping project” has also been granted UK Research Ethics Committee approval (18/LO/1169), including for collecting anonymized genotypic and phenotypic data from international collaborators. Informed consent for publication of clinical and molecular data was obtained from all patients included in this manuscript and/or their guardians.

## Results

### Subject Cohort

Twenty-seven individuals with *ATP1A3* variants in the DDD cohort (nearly 14,000 children recruited with their parents) were identified. In 7 individuals, the *ATP1A3* variant was classified as class 2 (likely benign) due to either an incongruent phenotype or high prevalence in healthy populations. For the remaining 20 individuals, 2 clinicians did not report back, 2 clinicians declined participation in the study, and 3 individuals did not consent to study participation. As a result, 13 individuals from the DDD cohort were included in this study. Another 11 individuals with class 4 and 5 (likely pathogenic/pathogenic) *ATP1A3* variants were volunteered by collaborating clinicians and included because they fulfilled the study criteria. Three were family members of 2 separate patients with DDD. Three individuals have been previously published in the literature (patients 3, 5, and 14). Table 2 summarizes the *ATP1A3* variants present in our cohort, including 13 novel variants.

**Table 2:**
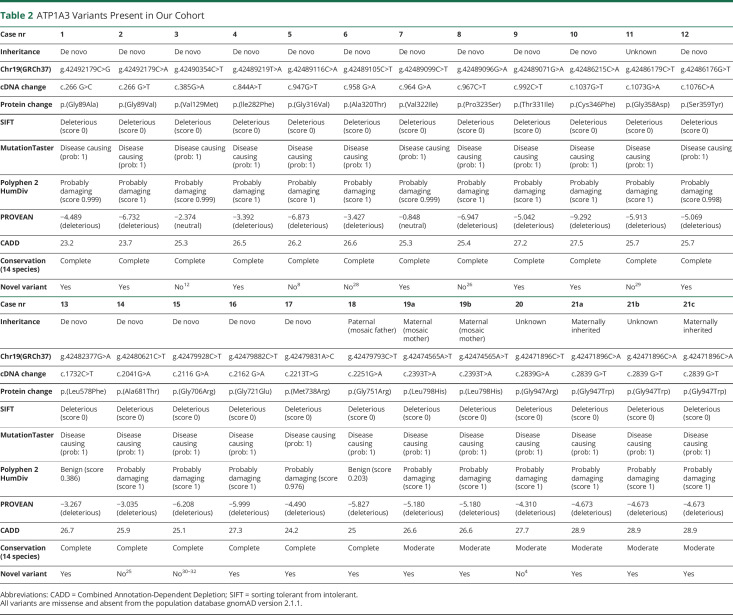
ATP1A3 Variants Present in Our Cohort

### Patient Age and Disease Onset

The median age of our cohort was 32.8 years (interquartile range [IQR] 15.5 years, range 3–52 years). Sixteen individuals (66.6%) were female. Age at onset of symptoms ranged from birth to 21 years with a median age at onset at 6 months (IQR = 9.94 months). The initial concern in order of frequency was developmental delay (n = 8, 33.3%), epileptic seizures (n = 6, 25%), dystonic events (n = 3, 12.5%), abnormal eye movements (n = 3, 12.5%), in 1 case accompanied by laryngomalacia and distal arthrogryposis all occurring at birth, ASD (n = 2, 8.33%), hemiplegic events (n = 1, 4.17%), and torticollis (n = 1, 4.17%). In 1 patient, the disease started after an encephalopathic episode, in 2 after febrile episodes.

### Paroxysmal Events

All individuals except for 2 (siblings 19a and 19b) had at least 1 type of paroxysmal event in their clinical history. On average, individuals had 2–3 different types of paroxysmal events. The most common were dystonic events (n = 14), followed by abnormal eye movements (n = 12) and epileptic seizures (n = 12). Hemiplegic episodes were less common in this cohort (n = 9). Autonomic episodes, such as tachycardia, mydriasis, and vomiting, were reported in 9 and apnoeic events in 4 patients.

### Epilepsy

Recurrent epileptic seizures were reported in 12 individuals. In 6 (50%), this was supported by epileptiform features on EEG. Seizure types were reported as focal seizures (n = 6), generalized tonic-clonic seizures (n = 5), and absence absence seizures, this is a seizure type seizures (n = 3). Four individuals had 2 seizure types, and 7 individuals had a history of status epilepticus (SE).

### Neurologic Features

Most individuals had complex neurologic phenotypes with 1–7 key neurologic features (median: 2.5; min: 0; max: 7; IQR: 2). Ataxia was the most frequently reported feature (n = 13), followed by dystonia (n = 10), spasticity (n = 8), microcephaly (n = 7), decreased muscle tone/hypotonia (n = 7), pyramidal signs (n = 5), dysarthria (n = 5), dysphagia (n = 4), chorea (n = 3), fluctuating muscle tone (n = 3), and increased muscle tone (n = 2). No individual had areflexia. Eight individuals reported worsening of their neurologic symptoms over time. Two individuals had no significant neurologic comorbidity.

### Neuroimaging

MRI was available in 18 individuals and abnormal findings reported in 11. These were most commonly cerebellar atrophy (n = 7, 38.9%), followed by hippocampal sclerosis (n = 3, 16.7%), cerebral atrophy (n = 2, 11.1%), thin corpus callosum (n = 2, 11.1%), and delayed myelination (n = 1, 5.6%). In 1 individual with MRI scans available at age 4 years and 11 years, the cerebellar atrophy progressed with age.

### Development

Cognitive impairment was reported in all individuals, but severity was extremely varied. Motor delay was reported in 20 (83.3%) individuals, with walking age ranging from normal at 13 months to some individuals not having learnt to walk by 18 years. Language delay was also reported in 20 (83.3%) individuals. Communication skills were very varied, ranging from starting to communicate at 9 months to not having acquired language at age 20 years. Grade of cognitive impairment was reported in 17 individuals and classified as mild in 5 individuals (29.4%), moderate in 5 individuals (29.4%), and severe in 7 individuals (41.2%). In 5 individuals (21%), regression of skills was reported, occurring either steadily over time (n = 3) or in association with a clear trigger (fever, infection, SE) (n = 2).

### Neuropsychiatry

Sixteen (66.7%) individuals had at least 1 neuropsychiatric diagnosis. 13 (54%) had behavioral difficulties, 7 (29%) had a diagnosis of ASD, 5 (21%) a diagnosis of ADHD, and 1 individual a diagnosis of COS.

### Symptom Combinations

**Figure 1:**
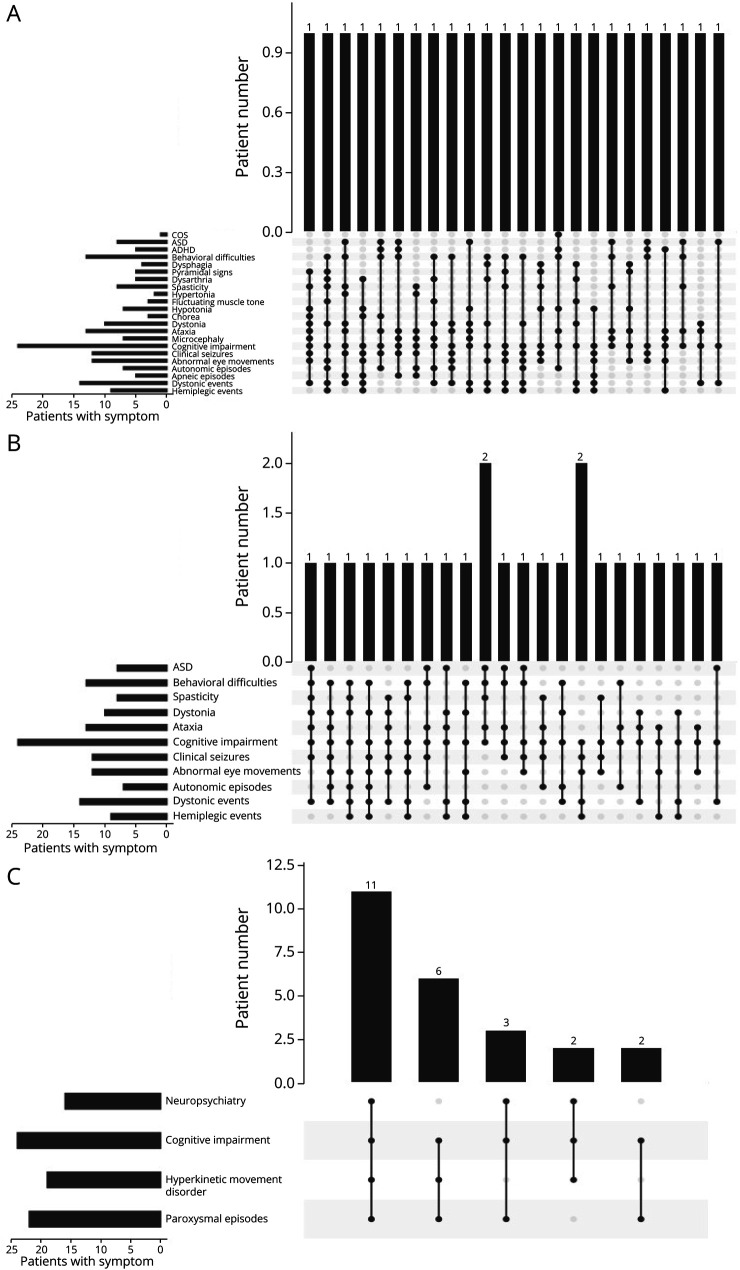
Symptom Combinations in Our Subject Cohort (A) Phenotypes in our cohort are extremely variable, with none of the patients sharing a combination of neurologic signs and symptoms. (B) Looking only at the most common 11 signs and symptoms, only 2 pairs of patients have overlapping features. (C) If we group symptoms into 4 categories: (1) neuropsychiatric symptoms, (2) hyperkinetic movement disorders, (3) paroxysmal episodes, and (4) cognitive impairment almost half our cohort (n = 11) has a phenotype combining all 4 categories and almost all individuals (22/24 individuals) have a phenotype combining 3 of the 4 categories.

Looking at the combination of symptoms in our 24 patients, phenotypes were extremely varied. Across all 22 neurologic signs and symptoms we collated, no 2 individuals shared the same combination (Figure 1A). Looking only at the 11 more common signs and symptoms, again there was little overlap, with only 2 pairs of individuals sharing the same features (Figure 1B). However, looking at the broader symptom categories, paroxysmal events, movement disorders, cognitive impairment, and neuropsychiatric symptoms, almost half of the cohort (45.8%, n = 11) had at least 1 symptom from each of all 4 categories, while most individuals (91.6%, n = 22) had at least 1 symptom from 3 categories (Figure 1C).

### Genotype-Phenotype Correlation in Individuals Sharing the Same *ATP1A3* Variant

Thirteen of the 21 variants reported in our cohort have not previously been published in the literature. In 3 individuals, the variants previously published refer to the same patients as in our cohort.^9,14,28^ The remaining previously published 5 variants (in different patients) are c.958G>A (p.Ala320Thr),^29^ c.967C>T (p.Pro323Ser),^30^ c.1073G>A (p.Gly358Asp),^31^ c.2116G>A (p.Gly706Arg),^32–34^ and c.2839G>A/C (p.Gly947Arg). Table 3 summarizes the individuals' phenotypes.

**Table 3:**
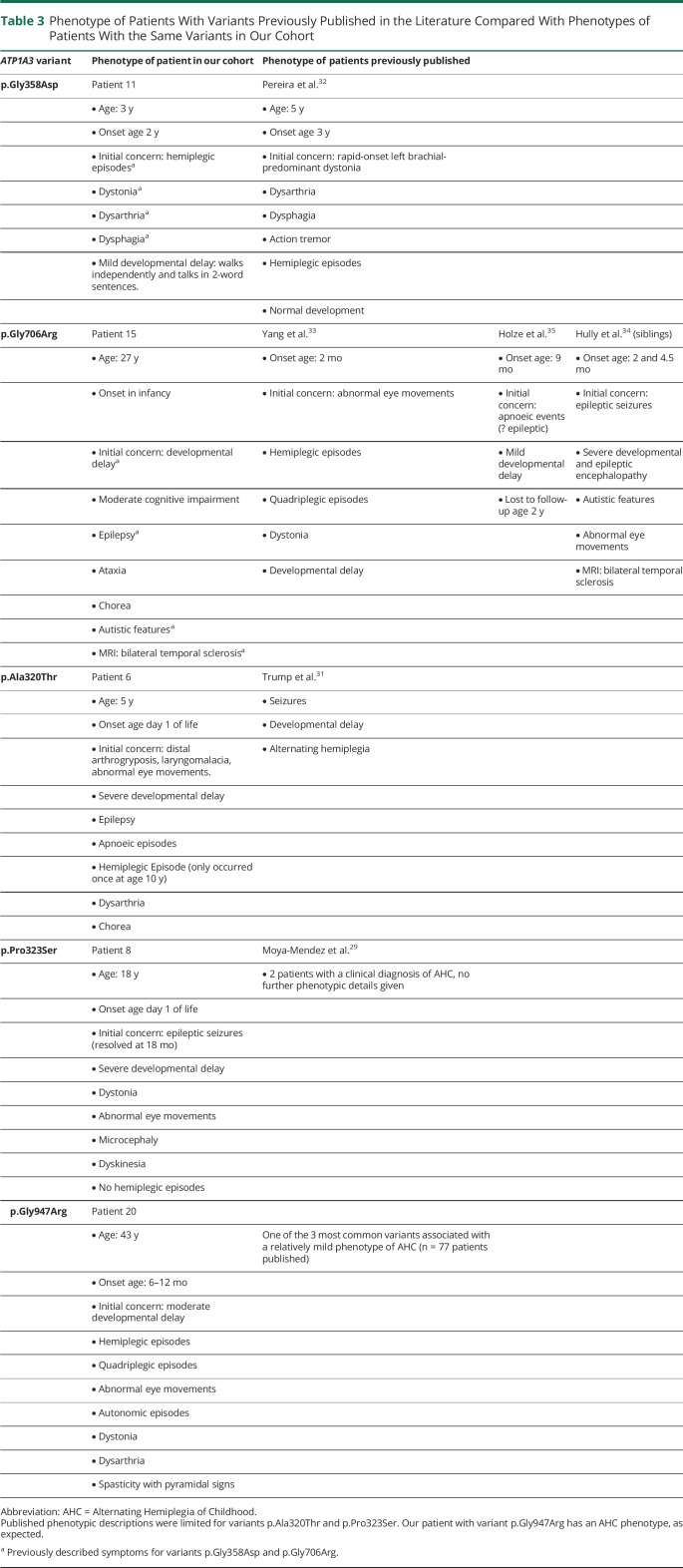
Phenotype of Patients With Variants Previously Published in the Literature Compared With Phenotypes of Patients With the Same Variants in Our Cohort

In our cohort, there are 2 families. They harbor 2 novel variants c.2393T>A (p.Leu798His) and c.2839G>T (p.Gly947Trp). The boy and girl (patients 19a and 19b), sharing the c.2393T>A (p.Leu798His) variant, have both inherited the variant from their mosaic mother. They have similar phenotypes, presenting with severe cognitive impairment, ataxia, and spasticity. Patient 19b developed secondary microcephaly. In our second family, a pair of brothers (21a and 21c) have inherited variant c.2839G>T (p.Gly947Trp) from their symptomatic mother (patient 21b). Individuals 21a and 21b both had a late onset presentation with dystonic episodes presenting at 14 and 21 years, respectively. Both are described as having mild cognitive impairment. The mother's neurologic phenotype has further deteriorated, and she is now ataxic and has nonparoxysmal dystonia. The older brother (patient 21c) has a much more severe phenotype, presenting much earlier at 6 months with torticollis and going on to develop hemiplegic episodes, dystonic episodes, abnormal eye movements, and epilepsy. He has moderate cognitive impairment.

### *ATP1A3* Variant Review

**Figure 2:**
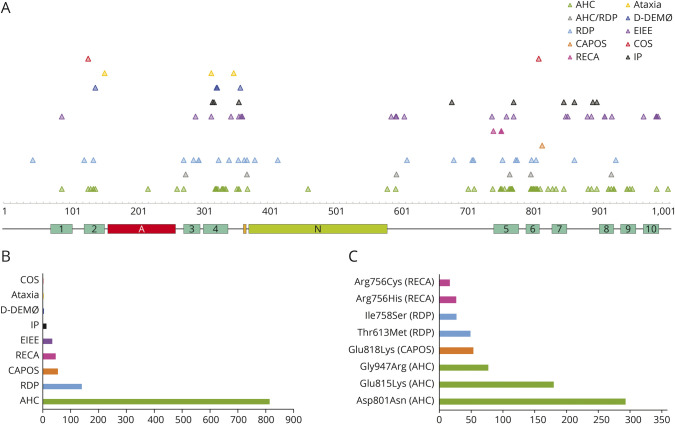
*ATP1A3* Variants (A) Distribution of variants across the α3 subunit of the sodium/potassium transporting ATPase. Location of protein domains is shown across the protein: turquoise, 1–10 transmembrane domains; red, actuator domain; orange, phosphorylation site; green, nucleotide binding site. Variants are color-coded per phenotype. (B) Frequency of phenotypes among the 1,000 + reviewed patients reported in the literature from 2004 to 2021. (C) Eight most frequent variants responsible for *ATP1A3-*related disorders in the 1,000 + reviewed patients reported in the literature from 2004 to 2021. Each variant leads to a specific phenotype. AHC = alternating hemiplegia of childhood; CAPOS = cerebellar ataxia, areflexia, pes cavus, optic atrophy, sensorineural hearing loss syndrome; COS = childhood onset schizophrenia; D-DEMØ = dystonia, dysmorphism of the face, encephalopathy with developmental delay, brain MRI abnormalities always including cerebellar hypoplasia, no hemiplegia (Ø), and neonatal onset of symptoms; EIEE = early infant epileptic encephalopathy; IP = intermediate phenotype; RDP = rapid-onset dystonia parkinsonism; RECA = relapsing encephalopathy with cerebellar ataxia.

The literature search yielded 349 publications. Of these, 134 reported individuals with heterozygous *ATP1A3* variants and included genotypic and phenotypic details and were included in this review. After translating all variants into transcript NM_152296, 168 different *ATP1A3* variants in total were found in the literature to date, corresponding to 1,108 reported patients. One hundred forty-four variants were missense variants, 15 small intragenic deletions (1 frameshift, 14 in frame), 2 small intragenic in frame deletion/insertions, 3 small intragenic in frame duplications, and 4 splice site variants. Reviewing the number of individuals published with each variant demonstrated that almost half the patients (42.8%) carry one of the 2 most common variants c.2401G>A (p.Asp801Asn) and c.2443G>A (p.Glu815Lys). Two-thirds (65.2%) of the cases (n = 721) carry one of 8 reported variants. These are c.2401G>A (p.Asp801Asn) (n = 293), c.2443G>A (p.Glu815Lys) (n = 180), and c.2839G>A/C (p.Gly947Arg) (n = 77), the 3 most common variants associated with AHC, and followed by c.2452G>A (p.Glu818Lys) (n = 53), the single-variant associated with CAPOS. Next in frequency are variants c.1838C>T (p.Thr613Met) (n = 49) and c.2273T>G (p.Ile758Ser) (n = 27), the most common genotypes associated with RDP, and finally, c.2267G>A (p.Arg756His) (n = 26) and c.2267G>T (p.Arg756Cys) (n = 16), the variants at the 756th amino acid (AA) residue causing RECA/FIPWE (Figure 2). The remaining 160 variants only account for 34.8% of the published individuals. For 84 variants, only 1 individual has been reported to date.

We attempted to assign phenotypes to all published cases. Mostly, this was already provided by the authors but when not, we used the information available in the manuscript to determine phenotype. If we were unable to assign 1 phenotype (e.g., if the patient had features of several *ATP1A3*-associated phenotypes), we used the term intermediate phenotype (IP). Although this approach is limited by the quality of published data, it nevertheless provided an overview of the most common *ATP1A3* phenotypes and associated genotypes. AHC was by far the most commonly reported phenotype, with 817 reported patients. A further 140 patients with RDP, 53 with CAPOS, 45 with RECA/FIPWE, 31 with EIEE, 13 with IP, 4 with D-DEMØ, 3 with isolated ataxia, and 2 with COS have also been reported (Figure 2). Variants were generally phenotype-specific, except for 6 variants (c.2305A>C [Thr769Pro], c.2767G>A [Asp923Asn], c.1109C>A [Thr370Asn], c.1790G>C [Arg597Pro], c.829G>A [Glu277Lys], c.2401G>T [Asp801Tyr]), which were associated with either RDP or AHC. The phenotypes CAPOS and RECA/FIPWE are mutation-specific, with all described patients with CAPOS carrying variant Glu818Lys and all except 1 patient with RECA/FIPWE carrying a variant involving AA residue 756.

### CADD Scores

**Figure 3:**
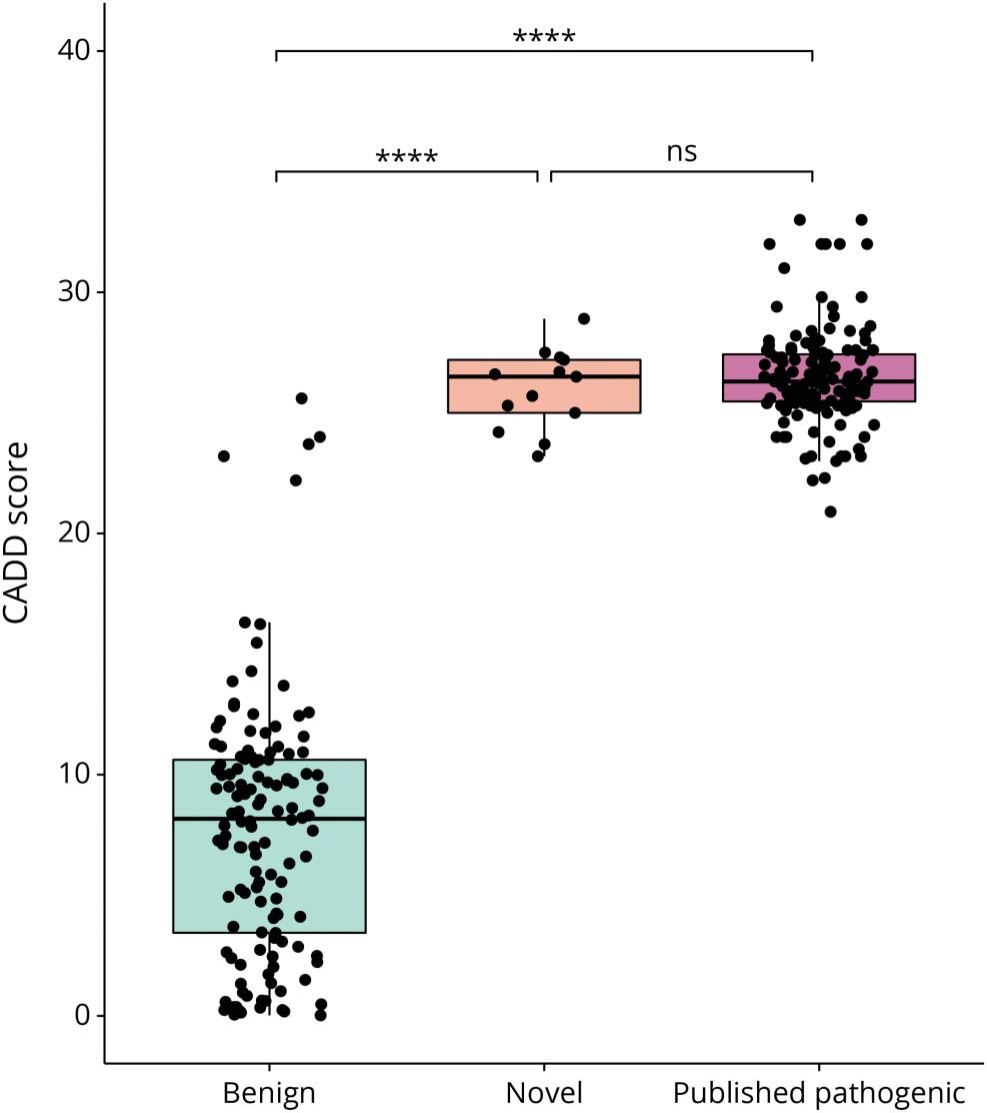
CADD Scores CADD scores associated with benign/likely benign *ATP1A3* variants published in ClinVar (green) are significantly lower than the novel *ATP1A3* variants in our patient cohort (orange) (*p* = 2.94e-39), and *ATP1A3* variants published as pathogenic in the literature (purple) (*p* = 1.05e-84). There is no significant difference between the novel and published pathogenic variants (*p* = 0.167). CADD = Combined Annotation-Dependent Depletion.

The mean CADD score for missense *ATP1A3* variants published in the literature as likely pathogenic/pathogenic was 26.5 (SD: 2.04). The mean CADD score for variants reported in ClinVar as likely benign/benign was 7.729 (SD: 5.27). This difference was statistically significant (*p* < 3.49e-85). The CADD scores of the novel variants identified in our patient cohort were similar to the variants published (mean: 25.98, SD: 1.65) (Figure 3).

### Constraint Analysis and Mutation Clusters

**Figure 4:**
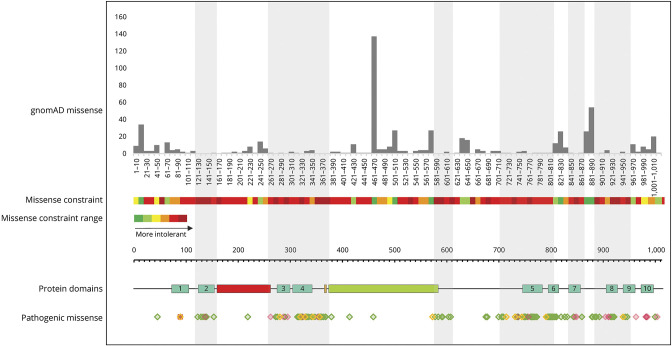
Constraint Analysis Missense allele counts for all *ATP1A3* missense variants were obtained from gnomAD v2.1.1. Missense amino acid substitutions are represented in grey (top section). We constructed a heatmap representing intolerance to missense changes, ranging from dark green through yellow, orange, and red with increasing intolerance (dark green = >20 variants, light green = 11–20 variants, yellow = 8–10 variants, light red 1–3 variants, dark red 0 variants). All pathogenic mutations from the literature (missense in green, small deletions/duplications in pink, and a sole frameshift mutation at residue 89 in red) and our cohort (all missense in yellow) and their distribution across the protein are shown (bottom section). The grey and white vertical shading represents mutation clusters and deserts, respectively. Highly constrained regions encompass transmembrane domains 2–9 (turquois), the phosphorylation site (orange), and the end of the nucleotide binding site (green), while the actuator domain (red) is situated in a mutation desert.

The most pathogenic and likely pathogenic variants identified both in the literature and this study lie within 6 clusters that correspond to benign missense variant deserts from the gnomAD database and vice versa (Figure 4). Benign missense constraint analysis identified gene deserts displayed as a heatmap. The 6 regions of constraint in which missense variation leads to pathogenicity are p.123-154, p.264-382, p.578-613, p.706-818, p.854-867, and p.887-955. These regions include key protein domains such as the transmembrane helices and the cytoplasmic P and N domains.

## Discussion

In this study, we describe the genetic and clinical features of a heterogeneous cohort of individuals with pathogenic or likely pathogenic variants in the *ATP1A3* gene. Previous studies have mostly recruited patients with a specific *ATP1A3*-related phenotype, such as AHC, RDP, CAPOS, or RECA, resulting in the description of clinically homogeneous cohorts. The starting point of this study differed in that patients were recruited based on the detection of *ATP1A3* variants within a WES study aimed at using advanced genomics to diagnose individuals with previously undiagnosed developmental delay. The resulting cohort is clinically very heterogeneous, apart from a degree of cognitive impairment present in all patients. Only 2 individuals in our cohort, that were recruited through a collaborator rather than the DDD study, met Aicardi diagnostic criteria for AHC, and no individual met diagnostic criteria for either RDP or CAPOS. We believe that our data set represents a growing reality commonly faced by clinicians: The increasing phenotypic pleiotropy associated with *ATP1A3* variants and identification of variants of uncertain significance poses a number of diagnostic challenges.

In addition to the broad phenotypic spectrum, our cohort was also genetically heterogeneous, with shared variants only seen among the members of the 2 participating families (individuals 19a and 19b and individuals 21a, 21b, and 21c). The genetic heterogeneity of the cohort might be reflective of its phenotypic heterogeneity because some phenotype-genotype association is evident for *ATP1A3*-related phenotypes. As discussed, all 53 CAPOS individuals published so far are linked to the variant Glu818Lys, and 45 published RECA/FIPWE cases are linked to variants involving substitution of arginine at residue 756. Within the AHC phenotype, it is also well documented that individuals with variant c.2443G>A (p.Glu815Lys) are more severely affected than individuals with variant c.2401G>A (p.Asp801Asn), while the third most common variant c.2839G>A/C (p.Gly947Arg) results in a milder phenotype.^35,36^ The 8 pathogenic variants mostly reported in the literature are associated with 1 specific phenotype (Figure 1).

Looking at the phenotypes of individuals sharing the same *ATP1A3* variant, it seems that while some genotypes are strongly associated with specific phenotypes, there are others that result in more phenotypic variability, such as those associated with c.2116G>A (p.Gly706Arg) and c.2839G>T (p.Gly947Trp). However, this is an observation based on a very small number of individuals, and it might be that given the opportunity to look at larger cohorts of the rarer *ATP1A3* variants, mutation-specific phenotypes will arise as they have for c.2452G>A (p.Glu818Lys) and variants at amino acid residue 756. This is important information to gather because it may help clinicians provide families with more accurate prognosis after diagnosis.

Fifty percent of our patients were reported to have a history of epileptic seizures. In half of these, epileptiform features were seen on EEG. The epilepsy phenotype varied among patients, with some having focal seizures, while others had generalized epilepsy. This variability in epilepsy phenotype has been described previously in a study of 51 patients with an AHC phenotype,^37^ where 32 individuals had an epilepsy diagnosis, 18 focal, and 11 generalized. Twenty-nine percent of our patients had a history of SE. Epilepsy treatment in this patient group is challenging. Many paroxysmal events are misdiagnosed as epilepsy and treated with antiepileptic medications with no benefit, whereas missing the epilepsy diagnosis in these patients might put them at risk of SE, which has been associated with severe aggravation of symptoms.^38^ It is important to correctly classify the events patients are experiencing and have a low threshold to conduct video-EEG investigations if uncertain of the nature of occurring events. This is especially important in individuals with a higher risk of a more complex epilepsy phenotype, such as those carrying variant c.2443G>A (p.Glu815Lys).

Earlier studies of patient cohorts with a clinical phenotype of AHC mostly reported normal MRIs.^39^ More recently, however, as the diverse phenotypes associated with *ATP1A3* are evolving, several reports of abnormal neuroimaging have also been published including cerebellar atrophy^40^ and PMG.^8–10^ Eleven of our patients also had abnormal MRI, most commonly with cerebellar atrophy, in 1 case proven to be progressive.

A large European cohort study of 155 individuals with a clinical diagnosis of AHC conducted in 2010 reached the conclusion that AHC is a nonprogressive disease.^41^ However, recent publications dispute this at least for some patients with an AHC phenotype.^38,42^ Little long-term data have been published for patients with other *ATP1A3*-related phenotypes. In our cohort, 21% (n = 5) of individuals had regression of skills over time. Although further longitudinal data are required, when counselling families, it may be important to discuss the risk of neurologic regression over time in *ATP1A3*-related disorders.

Traditionally, patients with an *ATP1A3*-related phenotype, such as AHC or RDP, have been diagnosed by using clinical diagnostic criteria.^43,44^ In recent years with the association of *ATP1A3* variants with a broadening clinical spectrum, this approach is not feasible for all patients because many do not fulfil classic phenotypic criteria. In addition, with broad genetic testing (gene panels, WES, and whole-genome sequencing) being brought into the diagnostic process at a much earlier stage, clinicians are often faced with an *ATP1A3* variant in an undiagnosed patient, trying to decide whether it is responsible for the phenotype, rather than having already reached a clinical diagnosis and using genetic investigations to confirm or inform it. We compared the phenotypic characteristics of our cohort with the diagnostic criteria published for AHC, RDP, CAPOS, and D-DEMØ (Table 1). Using the classic diagnostic criteria for AHC first introduced by Aicardi^45^ and subsequently used as inclusion criteria in clinical studies of patients with AHC,^41^ only 2 patients in our cohort and none of our DDD cohort achieve a clinical AHC diagnosis. Very recently, these criteria have been revised to include the presence of an *ATP1A3* variant and relaxed to describe a wider clinical spectrum of patients.^43^ Applying the new criteria, 1 patient recruited through the DDD study and another 7 patients of our extended cohort are clinically diagnosable with AHC. None of our patients met criteria for either RDP or CAPOS, and 1 patient met criteria for D-DEMØ. Overall, we found that most of our patients cannot be grouped into any of the existing described phenotypes. Rosewich et al.^46^ published major and minor criteria to support a diagnosis of an *ATP1A3*-related condition. The authors identified 5 major and 5 minor criteria for patients with infantile and early childhood onset, 6 major and 6 minor criteria for patients with childhood and adult onset, and 7 major and 7 minor criteria applicable for patients presenting at any age. Hence, for early onset, there are 12 major and 12 minor criteria overall, while for late onset, there are 13 major and 13 minor criteria. No cutoff is given as to how many criteria should be fulfilled to establish an *ATP1A3*-related condition diagnosis. In our cohort, 22 individuals had an onset in infancy/early childhood and 2 had an onset in later childhood or adulthood. All patients met at least 3 minor criteria. The only criteria met by all individuals in this cohort were cognitive impairment and negative family history or history suggesting autosomal dominant inheritance (both minor criteria). On average, patients met 3.4 major and 5 minor criteria. This approach of defining a spectrum of associated symptoms seems to be preferable for patients with *ATP1A3*-related disorders, if the threshold for the number of criteria needing to be fulfilled to prompt testing is kept low.

In addition to this, we found that in our cohort, looking for a combination of broad symptom categories, namely paroxysmal symptoms, hyperkinetic symptoms, neuropsychiatric symptoms, and cognitive impairment, rather than specific symptom combinations, was more likely to identify patients with *ATP1A3*-related disorder. A CADD score above 20 and a variant located within the mutation clusters in regions of constraint further support the diagnosis of an *ATP1A3*-related disorder.

There are limitations to our study. The phenotypic information was collected in retrospect through patient interview or case note review, rather than prospective evaluation. The phenotypic information available for published cases is variable and sometimes limited; patients are reported at different ages, clinical information is collected retrospectively, and different authors focus on different symptoms.

Two-thirds of all published individuals with *ATP1A3*-related disorders carry one of the 8 most common variants and display one of the 4 most common phenotypes: AHC, RDP, CAPOS, or RECA/FIPWE. However, the remaining third of individuals carry another 160 *ATP1A3* variants, and their phenotypes are very variable forming a phenotypic continuum of paroxysmal, neurologic, developmental, and neuropsychiatric features. Nowadays, clinicians are often faced with novel variants of uncertain significance in genes associated with rare diseases. Looking for a combination of paroxysmal events, hyperkinesia, neuropsychiatric symptoms, and cognitive impairment, as well as evaluating CADD score and variant location, can aid in the diagnosis of an *ATP1A3*-related disorder. The ongoing collection of phenotypic information of individuals carrying rarer variants will help us discover further mutation-specific phenotypes and aid in disease prognosis and management.

## GLOSSARY

ADHD: attention-deficit and hyperactivity disorder
AHC: alternating hemiplegia of childhood
ASD: autism spectrum disorder
CADD: Combined Annotation-Dependent Depletion
CAPOS: cerebellar ataxia, areflexia, pes cavus, optic atrophy, sensorineural deafness
COS: childhood onset schizophrenia
DDD: Deciphering Developmental Disorders
EIEE: early infancy epileptic encephalopathy
FIPWE: fever-induced paroxysmal weakness and encephalopathy
IP: intermediate phenotype
NKA: sodium-potassium-ATPase
PMG: polymicrogyria
RDP: rapid-onset dystonia parkinsonism
RECA: relapsing encephalopathy with cerebellar ataxia
SE: status epilepticus
SIFT: sorting tolerant from intolerant
VUS: variants of uncertain significance
WES: whole-exome sequencing
